# Improving naltrexone compliance and outcomes with putative pro- dopamine regulator KB220, compared to treatment as usual

**DOI:** 10.15761/JSIN.1000229

**Published:** 2020-05-30

**Authors:** Kenneth Blum, Lisa Lott, David Baron, David E Smith, Rajendra D Badgaiyan, Mark S Gold

**Affiliations:** 1 Western University Health Sciences, Graduate College, Pomona, CA, USA; 2 Division of Behavioral Precision Management, Geneus Health, LLC, San Antonio, TX, USA; 3 Department of Pharmacology, University of California San Francisco School of Medicine, San Francisco, CA, USA; 4 Department of Psychiatry, Icahn School of Medicine Mt Sinai, New York, NY, USA and Department of Psychiatry, South Texas Veteran Health Care System, Audie L. Murphy Memorial VA Hospital, San Antonio, TX, Long School of Medicine, University of Texas Medical Center, San Antonio, TX, USA; 5 Department of Psychiatry, Washington University School of Medicine, St. Louis, Mo, USA

**Keywords:** opioid crisis, agonist vs antagonist therapy, naltrexone, vivitrol®, kb220, pro-dopamine regulation, combination therapy of naltrexone and kb220

## Abstract

A recent analysis from Stanford University suggested that without any changes in currently available treatment, prevention, and public health approaches, we should expect to have 510,000 deaths from prescription opioids and street heroin from 2016 to 2025 in the US. In a recent review, Mayo Clinic Proceedings (October 2019), Gold and colleagues at Mayo Clinic reviewed the available medications used in opioid use disorders and concluded that in private and community practice adherence is more important as a limiting factor to retention, relapse, and repeat overdose. It is agreed that the primary utilization of known opioid agonists like methadone, buprenorphine and naloxone combinations, while useful as a way of reducing societal harm, is limited by 50% of more discontinuing treatment within 6 months, their diversion, and addiction liability. Opioid agonists may have other unintended consequences, like continuing the down regulation of dopamine systems. While naltrexone would be expected to have opposite effects, adherence is also low even after detoxification and long acting naltrexone injections. Recent studies have shown Naltrexone is beneficial by attenuation of craving via “psychological extinction” and reducing relapse. Buprenorphine is the MAT of choice currently but injectable Naltrexone plus an agent to improve dopaminergic function and tone may renew interest amongst addiction physicians and patients. Understanding this dilemma there is increasing movement to opt for the non-addicting narcotic antagonist Naltrexone. Even with extended injectable option there is still poor compliance. As such, we describe an open label investigation in humans showing improvement of naltrexone compliance and outcomes with dopamine augmentation with the pro- dopamine regulator KB220 (262 days) compared to naltrexone alone (37days). This well studied complex consists of amino-acid neurotransmitter precursors and enkephalinase inhibitor therapy compared to treatment as usual. Consideration of this novel paradigm shift may assist in not only addressing the current opioid epidemic but the broader question of reward deficiency in general.

## Introduction

In response to the devastating and unimaginable death toll of hundreds of thousands of people dying from overdose of opioid/ opiate narcotics throwing many communities in economic trouble, the addiction medicine field is in a panic [1]. Specifically, a recent analysis from Stanford University suggested that without any changes in currently available treatment, prevention, and public health approaches, we should expect to have 510,000 deaths from prescription opioids and street heroin from 2016 to 2025 in the US. The primary utilization of known opioid agonists like methadone, buprenorphine and naloxone combinations, while useful as a way of reducing societal harm, is limited by their abuse and addiction liability [2]. However, there is increasing movement to opt for the non-addicting narcotic antagonist naltrexone. While this seems to be an important option the current evidence related to its benefits and outcomes requires improvement. One major issue in treatment is poor compliance.

Moreover, the epicenter of the second but worst opioid epidemic driven in-part by Big Pharma (now being fined) with disastrous deaths due to overdose is so overwhelming the total societal cost is reaching an unimaginable amount north of one –trillion. This epidemic has crippled so many communities across America with dismal outcomes in spite of utilization of MAT such as buprenorphine combinations. There is argument that one reason for failure is underutilization, in-part due to high addiction liability. Moreover, simply the idea of treating one narcotic with another narcotic, even with some special properties including partial agonism at Mu receptors, seems counter intuitive [3].

Understanding the nature of addiction liability has led to the increasing utilization of narcotic antagonism. One –major problem is compliance and as such the long-acting naltrexone injectable (e. g. Vivitrol®) has been developed with varying results. One issue is the misbelief that naltrexone molecules actually block opioid craving behavior via direct neurobiological mechanisms. This fallacy has led to false claims of the benefits of narcotic antagonism. We hereby point out that in fact the primary benefit is simply “psychological extinction.” Understanding the psychopharmacological profile mandates the continued search for better treatments including the induction of genetically guided (GARS) precision pro-dopamine regulation and subsequent potential induction of dopamine homeostasis [4]. The latter is a more laudable goal for clinicians to have in the treatment /clinical toolbox, and while requiring additional research, may offer promise.

The history of MAT goes back to the 60’s when Dole & Nyswander [5] suggested the use of methadone, a full opioid agonist at mu receptors, as a maintenance therapeutic approach. This maintenance approach was followed up with buprenorphine, a partial agonist whereby this medication has high affinity for the mu opioid receptor (MOR) but has an upper limit or “ceiling” on maximal opioid effects. However, over 60 years ago, naloxone was synthesized and patented and subsequently produced by Endo Labs as a narcotic antagonist able to act as an antidote to opioids. Naloxone was adopted by Yale New Haven Hospital’s emergency department over 40 years ago [6]. Similar to naloxone, the long-acting naltrexone is a MOR antagonist. It was first synthesized in 1963 by Endo Laboratories, which was later purchased in 1969 by DuPont Pharmaceuticals. Though the drug remained essentially dormant for several years, it attracted interest in 1972 when Congress passed the Drug Abuse Office and Treatment Act for the purpose of developing non-addictive (i.e., non-agonist) treatments for heroin addiction. It is noteworthy, that in the early 70s the National Institute on Drug Abuse (NIDA) approached Endo Laboratories to augment their clinical research to obtain FDA approval for opioid addiction and even alcoholism [7–10]. One of the first to implicate the potential of naltrexone to treat opioids was A. Goldstein [11] and later clinical trials from a number of USA scientists [12–15]. Even before early clinical trials related at first to alcoholism by O’Brien and Volpicelli [16], evidence from Blum’s group showed an unexpected blocking of not only ethanol induced sleep -time in mice [17] but also inhibiting chronic ethanol dependence using the Goldstein Inhaler method [18].

### Compliance a major issue

It is to be noted that the antagonistic approach is represented by naltrexone. In 1985, the oral version was approved by the FDA. A major advantage is that while naltrexone occupies the opioid type receptors especially mu, it does not produce euphoria or reward. In terms of the pharmacokinetics, the oral version requires daily or three times weekly administration, but patients can relapse simply by stopping the medication for 48 hours. As such the oral version has had only limited success. However, an extended release version of naltrexone has become available which may prevent relapse up to 30 days. While some patients find it worthwhile and convenient to return monthly for an injection rather than to take a daily medication, many do not. In favor of the injectable form sold under the name of Vivitrol® (XR-NTX), McDonald *et al.* [19] in a clinical trial of this form in the probation system found, those randomly assigned to 6 months on extended release naltrexone had significantly more drug-negative urines and a lower relapse rate than patients given usual treatments in the community.

It is noteworthy to know that naltrexone is a relatively weak antagonist of κ- and δ-receptors and a potent μ-receptor antagonist. Dosages of naltrexone that effectively reduce opioid and alcohol consumption also strongly block μ-receptors, but down-regulates meso-limbic dopamine release. While these studies show benefit especially in the short term there is ongoing evidence that the retention and compliance on Vivitrol® is not sufficient to characterize adherence as high [20,21] Specifically in a meta –analysis, of randomized, controlled trials, only 3 (14%) met criteria for high levels of adherence assurance, 5 (23%) met medium adherence assurance criteria, and 14 (64%) met low adherence criteria. Moreover, the Spearman correlation between risk ratios for return to heavy drinking (for naltrexone vs. placebo) and the level of adherence assurance (low vs. medium vs. high) was significant (r=−.62, p=.025). The completion of the study of opioid treatment with extended release Vivitrol (XR-NTX) was associated with superior outcomes and less likely relapse (defined as daily use), with a much greater time to relapse despite higher rates of concurrent non-opioid substance use like cocaine. In terms of long-term extended release injectable (XR-NTX) for opioid dependence there was a higher compliance in Opioid Use Disorder (OUD) than for Alcohol Use Disorder (AUD), but after completion of study most participants discontinued treatment with XR-NTX largely due to “feeling cured” and “wanting to do it on my own” rather than external barriers such as cost or side effects [21]. It is imperative then, that other modalities in combination with Vivitrol® should be considered. While one barrier of its use is that detoxification of the patients is required before an antagonist can be administered, key opinion leaders believe that the greatest hurdle has to do with improving naltrexone compliance and outcomes.

We now provide a detailed analysis of a previous hypothesis type article showing some dramatic and clear evidence that by coupling a known highly researched pro-dopamine regulstor, KB220, a complex of amino-acid neurotransmitter precursors and enkephalinase inhibitor therapeutic to long-term methadone addicts rapidly detoxed with naltrexone (oral form) improved compliance and outcomes [22]. The complex KB220 and variants over a 50 year sojourn, displays at least forty-one studies related to its drug and non-drug addictive benefits in terms of pre-clinical and human clinical trials [23].

### Rationale of investigation

It is noteworthy, that Against Medical Advice (AMA) rate (the rate at which patients or addicts leave treatment before treatment goals are reached) among hardcore addicts even today approaches as high as 90%. The basic concept of *“rapid detoxification method”* is to provide the patient with a pure narcotic antagonist to eliminate by blocking the opiate induced pleasurable effects. However, while the addiction medicine or recovery space embraces this approach, it is rift with poor compliance and still significantly high recidivism rate [20,21]. One reason as expressed by many scientists especially Sinclair’s group in Helsinki [24] is that in spite of claims of naltrexone directly blocking craving behavior for opioids and even alcohol, based on its pharmacological profile and experiments thereof this drug and narcotic antagonists in general has little effect on craving behavior. The clinical reduction in craving behavior is simple due to “psychological extinction.” In fact Kirchmayer *et al.,* [25] following a systematic review on the efficacy naltrexone maintenance (oral), suggested that there was no significant evidence to support the utilization of naltrexone maintenance in the treatment of Opioid Use Disorder (OUD).

## Methods and materials

### Subjects

The proposed combination therapy of rapid detoxification using oral naltrexone (Trexan®) alone and in combination with the Pro-dopamine regulator KB220 with heavy addicted long-term methadone patients, was accomplished at the *J.T. Payte MD, PA Clinic*, San Antonio, Texas. Inclusion criteria for study entry included both genders with a history of up to 30 years abusing psychoactive chemicals including opioids. Prior to entry each patient was diagnosed as hardcore addicts using the DSM-1V criteria for opioid dependence/heroin. The total number of study participants was 1012 mixed gender divided into two unbalanced groups consisting of Group BC (baseline control) and Group ENTX (experimental with naltrexone). Group BC consisted of 1000 patients (N=700 M; N=300 F) and Group ENTX consisted of 12 patients (N-9 M; N=3 F). The average age of the total population was 49 whereby the age range was from 40–70 years. The trial received IRB approval from not only the San Antonio Methadone Clinic but from PATH Medical Foundation (registration #IRB00002334). In addition each study participant signed an IRB approved consent form prior to their entry into the investigation (demographic Table 1).

### Protocol for rapid detoxification

In this investigation our rapid detoxification method was consistent with every participant, whereby, each subject (N=1012), was pre-evaluated by first administering an injection of 0.4 to 0.8 mg of naloxone (Narcan®) and if they passed this first test they were subsequently delivered an oral dose of 2.5 mg of naltrexone (Trexan®). Following the naltrexone dose each subject was re-evaluated for withdrawal symptoms over a 90 minute period. Finally, if they passed this second test they were then provided with 50 mg of oral naltrexone. The 1012 patients were given 50 mg of naltrexone at the clinical site daily until the patient relapsed. Of cause following the initial pre-evaluation the 12 patients placed in the ENTX group that were selected, had been maintained on methadone on the average of 18–30 years.

### Precursor amino-acid and enkephalinase inhibition therapy

The basic formula for this study conducted in the early 90’s primarily consisted of varying amounts of L-phenylalanine (precursor to dopamine synthesis in brain); -Tyrosine (rate limiting molecule for dopamine synthesis), L-Tryptophane (precursor for serotonin synthesis in brain); chromium salt (increases gut to brain tryptophane for serotonin synthesis in brain); L-Glutamine (precursor for GABA synthesis in brain), D-phenylalanine (brain enkephalinase inhibitor) and pyridoxine-5 –phosphate (a enzymatic catalyst). The research code name is KB220/KB220Z and to date there are at least 41 published studies including pre-clinical and human (see reference [23] for a review of all studies to date). Over a 40 year sojourn while the basic formula has stayed the same, however, Blum’s group have altered the ingredients as new facts suggested inclusion such as N-Acetyl – Cysteine (NAC) and Rhodiola among other important ingredients like NADH. One outcome measure was simply the number of days without a relapse or self-report of refusal to take either the naltrexone alone or in combination with the amino-acid formula was counted. Moreover, lack of relapse was also identified by a routine urine Drug –Tox screen (PharmChem –San Francisco). It is noted that albeit some failure, each patient was evaluated on a daily basis either via phone or face to face contact.

### Statistics

In this investigation we utilized a simple Fischer student –t-test with an 95% confidence and an alpha at 0.05 for statistical significance between group BC (N-1000) and group ENTX (N=12). We also used the Satterthwaite approximation which is away to account for two different sample variations to correct for unequal variances. We used the following formula:

$$\text{Se}=\sqrt{\left(\text{s}12/\text{n}1+\text{s}22/\text{n}2\right)}.$$

## Results

**Figure 1.**
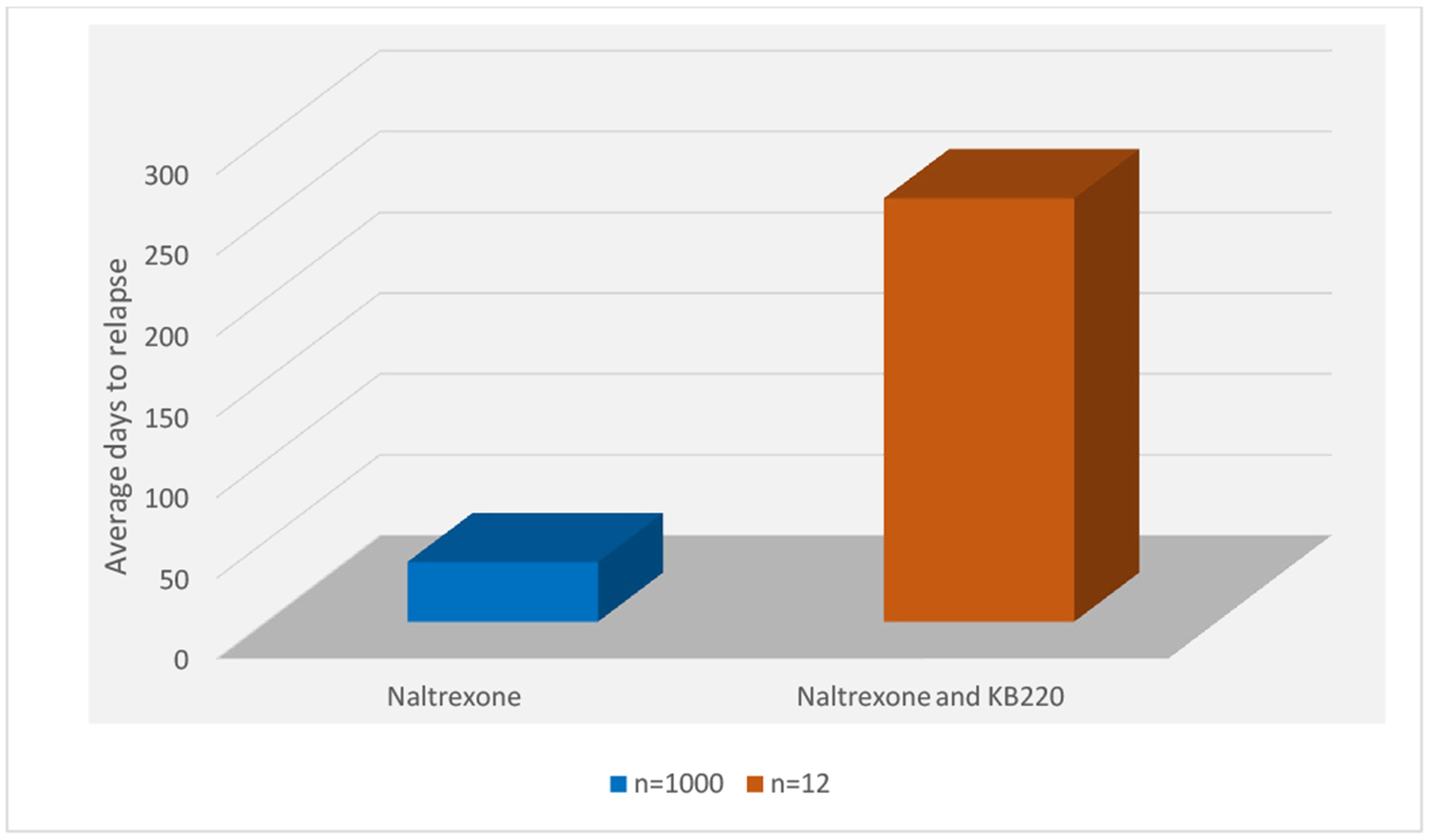
Rapid detoxification of Methadone patients with Naltrexone (N=1000) vs. Naltrexone and KB220 (N=12)

Given the complexity of monitoring 1,012 patients daily for approximately 365 days (study truncated), the staff directed by Dr. J.T. Payte (now deceased), albeit a few failures, carefully documented the results of the investigation. As displayed in Figure 1, the results were very dramatic showing a highly significant enhancement of compliance when we coupled the rapid detoxification procedure with the KB220 complex. Specifically, the J.T. Payte Clinic of San Antonio, Texas staff calculated for the BC group of 1,000 without the KB220 the average number of days of compliance without KB220 and found it to be only 37 ± 7.7 SE days. In comparison of treating with the addition of daily administration of KB220 the dozen patients tested (combination of naltrexone plus KB220) was relapse-free for an average of 262 ± 16.4 SE days. Statistical analysis revealed high significance in favor of the naltrexone + KB220 combination compared to naltrexone alone with a P < 0.0001 @ 95% confidence (Figure 1).

## Discussion

The coupling of amino-acid therapy and enkephalinase inhibition, while blocking the delta-receptors with a narcotic antagonist even if weak, may be a quite promising novel method to not only induce rapid detox in chronic methadone patients but as a frontline modality to treat OUD. This may also have important ramifications in the treatment of both opiate and alcohol-dependent individuals; enhanced compliance with Vivitrol® (as an extended release injectable) and especially as a relapse prevention tool.

### Naltrexone and dopamine release

It may also be interesting to further test this hypothesis both in a more substantial cohort and with the sublingual combination of the partial opiate mu receptor agonist buprenorphine. In terms of buprenorphine and dopaminergic function, acute doses increase dopamine release, whereas, chronic administration leads to reduced dopamine release. However, with naltrexone it was found that in human’s dopamine release increased over an 8-day period but dissipated over time. In animal studies the opioid antagonist naltrexone has been shown to attenuate the subjective effects of amphetamine. However, the mechanisms behind this modulatory effect were unknown up until April 2017, when Nitya Jayaram-Lindström and associates [26] hypothesized that naltrexone would diminish the striatal dopamine release induced by amphetamine, which is considered an important mechanism behind many of its stimulant properties. They used positron emission tomography and the dopamine D2-receptor radioligand [11C] raclopride in healthy subjects to study the dopaminergic effects of an amphetamine injection after pretreatment with naltrexone or placebo. In a rat model, they used microdialysis to study the modulatory effects of naltrexone on dopamine levels after acute and chronic amphetamine exposure. In healthy humans, naltrexone attenuated the subjective effects of amphetamine, confirming previous results. Amphetamine produced a significant reduction in striatal radioligand binding, indicating increased levels of endogenous dopamine. However, there was no statistically significant effect of naltrexone on dopamine release. The same pattern was observed in rats, where an acute injection of amphetamine caused a significant rise in striatal dopamine levels, with no effect of naltrexone pretreatment. However, in a chronic model, naltrexone significantly attenuated the dopamine release caused by the reinstatement of amphetamine.

Collectively, these data suggest that the opioid system becomes engaged during the more chronic phase of drug use, evidenced by the modulatory effect of naltrexone on dopamine release following chronic amphetamine administration. The importance of opioid-dopamine interactions in the reinforcing and addictive effects of amphetamine is highlighted by these findings and may help to facilitate medication development in the field of drug dependence especially as it also relates to buprenorphine /naloxone combinations.

## Future perspective

It is a fact that most of the FDA approved drugs work by favoring dopamine blockade and subsequent extinction of substance seeking behavior with full or partial agonistic activity (e.g. Methadone & Buprenorphine). We also know that thses approved FDA MAT have high addiction liability and in the case of methadone even cardiovascular adverse effcts. However, if we could find novel ways to improve both the compliance and outcomes with the use of naltrexone, in the injectable form, as we show herein, the recovery cimunity may be well served.

Along these lines, we are not surprised about our dramatic findings with KB220. Resting fMRI data analysis in the heroin users after KB220 and placebo clearly reveal that KB220 induced an increase in BOLD activation in caudate-accumbens-dopaminergic pathways compared to placebo following 1-hour acute administration. Furthermore, KB220 also reduced resting-state activity in the cerebellum of abstinent heroin addicts suggesting an induction of dopamine homeostasis. In the second phase of this pilot study of all 10 abstinent heroin-dependent subjects, Blum *et al.* [27] observed that three brain regions of interest were significantly activated from resting state by KB220 compared to placebo (p < 0.05). Increased functional connectivity was observed in a putative network that included the dorsal anterior cingulate, medial frontal gyrus, nucleus accumbens, posterior cingulate, occipital cortical areas, and cerebellum [27].

As pointed out by Kunøe *et al.* [28] naltrexone, similar to other medications such as methadone and buprenorphine, shows some success, especially with the narcotic antagonistic approach used in the treatment of OUD. However, compliance is a real barrier of prolonging the benefit of “psychological extinction” possibly by balancing dopamine with KB220. This could have futuristic therapeutic value. We are suggesting that since the addiction process either linked to DNA polymorphic risk alleles or epigenetic insults effecting normal mRNA transcription, is a highly complex disorder involving multi-neurotransmitter pathways, pharmaceutical singular targets on the opioid system seems too reductionist. Instead, we are hereby suggesting that targeting the entire array of neurotransmitter networks with KB220 seems prudent. The one hit approach as indicated with MAT, either agonistic or antagonistic is not a panacea and we must continue to find mores neuroscience based sophisticated solutions. One important direction involves ways to affect resting state functional connectivity, which may serve as the ideal tool to study brain changes *in vivo*, as is proposed by the NIDA ABCD study [29]. This is now underscored and observed with KB220 in naive rodents and heroin addicts [27, 30]. Moreover, we must also consider better neurogenetic based risk diagnostic early identification for prophylaxis as discussed by Blum & Baron [30].

To be clear there may be other promising modalities other than MAT such as repetitive transcranial magnetic stimulation (rTMS), [31] exercise [32] and even new medications with positive allosteric modulators of GABA-A receptors [33].

## Conclusion

Naltrexone holds some promise in the short term as a psychologically induced deterrent therapeutic modality. Moreover, by adding a pro-dopamine regulator to help balance dopaminergic function especially important reward circuitry sites, has heuristic value. Therefore, these findings presented herein should be embraced by the clinical community challenged with early harm reduction in active OUD patients.

Furthermore, we must be reminded that drug seeking behavior is indeed a chronic enduring illness that has genetic antecedents and there is no real quick fix [34]. Instead, the long-term recovery goal while it includes abstinence, most importantly, eliminating the unwanted “white-knuckle sobriety” and replacing it with a better quality of life, may reside in the induction of dopamine homeostasis. With this tenant in mind as suggested by Srivastava and Gold [35], “*only then will we be able to consistently and effectively address not only the opioid epidemic but the broader question of addiction as a whole”.* Similarly for a review of the area the reader is encouraged to see a number of earlier published works on narcotic antagonism and buprenorphine [2,34–46] With all this stated, we are hereby cognizant that possibly at much lower doses NTX (oral) may have some analgesic properties which potentially as a feedback mechanism may induced enkephalin release a novel proposition.

## Figures and Tables

**Table 1.** Demographics (BC=baseline control; ENTX=experimental with naltrexone)

Participant groups	Group BC	Group ENTX
Male	N=700	N=9
Female	N=300	N=3
Average Age	49	
age range	40–70 years.	
